# The *Drosophila* Gene *RanBPM* Functions in the Mushroom Body to Regulate Larval Behavior

**DOI:** 10.1371/journal.pone.0010652

**Published:** 2010-05-14

**Authors:** Nadia Scantlebury, Xiao Li Zhao, Verónica G. Rodriguez Moncalvo, Alison Camiletti, Stacy Zahanova, Aidan Dineen, Ji-Hou Xin, Ana Regina Campos

**Affiliations:** Department of Biology, McMaster University, Hamilton, Ontario, Canada; Queensland Brain Institute, Australia

## Abstract

**Background:**

In vertebrates, *Ran-Binding Protein in the Microtubule Organizing Center* (*RanBPM*) appears to function as a scaffolding protein in a variety of signal transduction pathways. In *Drosophila*, *RanBPM* is implicated in the regulation of germ line stem cell (GSC) niche organization in the ovary. Here, we addressed the role of *RanBPM* in nervous system function in the context of *Drosophila* larval behavior.

**Methodology/Principal Findings:**

We report that in *Drosophila, RanBPM* is required for larval feeding, light-induced changes in locomotion, and viability. *RanBPM* is highly expressed in the Kenyon cells of the larval mushroom body (MB), a structure well studied for its role in associative learning in *Drosophila* and other insects. *RanBPM* mutants do not display major disruption in nervous system morphology besides reduced proliferation. Expression of the *RanBPM* gene in the Kenyon cells is sufficient to rescue all behavioral phenotypes. Through genetic epistasis experiments, we demonstrate that *RanBPM* participates with the *Drosophila* orthologue of the Fragile X Mental Retardation Protein (FMRP) in the development of neuromuscular junction (NMJ).

**Conclusions/Significance:**

We demonstrate that the *RanBPM* gene functions in the MB neurons for larval behavior. Our results suggest a role for this gene in an FMRP-dependent process. Taken together our findings point to a novel role for the MB in larval behavior.

## Introduction

The fruitfly *Drosophila melanogaster* has been employed successfully as a genetically tractable model for the study of nervous system development and function. A large fraction of behavioral studies in *Drosophila* have focused on the adult fly. However, the relative complexity of the adult nervous system makes the identification of neural networks involved in these behaviors challenging. More recently, the *Drosophila* larva has emerged as a simpler model for the identification of the neural circuitry underlying behaviors such as learning and memory [1], [2] and feeding [3], [4].

The *Drosophila* foraging larva lives in the food source. It feeds constantly until it wanders off the food substrate, in search for a site to undergo metamorphosis. Consistently, throughout the foraging stage of larval development *Drosophila* is repelled by light and continuously attracted to food. As the larva enters the wandering stage food intake ceases, the gut is emptied and it becomes aversive to food and indifferent to light [5], [6].

We took a genetic approach toward the identification of neurons that play a role in the control of locomotion. Control of locomotion was studied in the context of light induced changes to larval movement that reflect the *Drosophila* larva's repulsion from light. This approach led to the identification of a transposable element mutation that caused severe disruption in larval response to light as measured in this assay as well as in locomotion, feeding behavior, and lethality. The P-element insertion disrupts the *Drosophila* orthologue of the vertebrate *Ran-Binding Protein in the Microtubule Organizing Center* (*RanBPM*) gene, originally identified in a yeast two-hybrid screen using Ran GTPase as bait [7]. Function of *RanBPM* in Ran-dependent processes and in the centrosome has not been established. In *Drosophila*, *RanBPM* is required in the ovary for the regulation of germ line stem cell (GSC) niche organization [8].

RanBPM protein contains multiple conserved domains implicated in protein-protein interactions such as a SPRY domain found Sp1A and ryanodine receptors, a lyssenchephaly homology (LisH) motif, a motif C-terminal to LisH (CTLH), and a CRA (CT11-*RanBPM*) domain. RanBPM binds to and regulates the function of a variety of proteins e.g. [9], [10], [11], [12], [13], [14], [15]. The functional relevance of several of these interactions is yet to be established. Of interest is the reported RanBPM-mediated regulation of TrkA and PlexinA receptors [16], [17]. Collectively these observations suggest a role for RanBPM as a scaffolding protein.

One potential partner of RanBPM, as established by in vitro binding assays, is the Fragile X Mental Retardation Protein (FMRP)[18]. Lack of FMRP function is the underlying cause of the more prevalent form of inherited mental retardation [19]. FMRP is an RNA binding protein, highly conserved and required for mRNA transport and translational suppression in the context of synaptic plasticity. In *Drosophila* as well as other model systems, lack of the *Drosophila* orthologue gene (*dfmr1*) causes excess synaptic elaboration [20], [21], [22] and disruption in activity-dependent plasticity [23], [24].

Using a number of different genetic and cell biology tools we demonstrate that in *Drosophila RanBPM* function is required in the nervous system for the modulation of locomotion by light, feeding behavior and growth. *RanBPM* is highly expressed in the Kenyon cells of the mushroom bodies (MB) and targeted expression in these neurons is sufficient to rescue all behavioral phenotypes of the mutant larvae. *RanBPM* mutations do not appear to disrupt basic aspects of nervous system development.

The MB is a prominent neuropil structure implicated in olfactory learning and memory, as well as other complex behaviors of the adult fly (e.g. reviewed by [25], [26]). MB function in larval behavior has not been as extensively studied. Recent investigations indicate a role for MB output in a model for associative learning in the third instar larva e.g. [1], [27].

As a first step toward the identification of the signal transduction pathways supported by *RanBPM* function, we sought evidence for an interaction with proteins previously identified, in vertebrates, as potential partners of *RanBPM*. We found that reduction of *RanBPM* function suppresses the neuromuscular junction (NMJ) overgrowth phenotype caused by mutations in the *Drosophila* orthologue of FMRP (*dFmr1*) suggesting that RanBPM protein may contribute to FMRP-dependent processes. Taken together our results demonstrate that *RanBPM* function in the MB contributes to the regulation of larval behavior and suggest a novel role for this structure.

## Materials and Methods

### *Drosophila* strains and culture

Synchronized larvae at the early third instar foraging stage were obtained as described [6] and grown at 25°C unless otherwise stated, in light/dark cycles in food supplemented with vitamin A (1.25 g/L). Mutations were kept over a *CyO*(*y* ^+^) balancer in a *yw* background for the identification of homozygous mutants by the mouth hook phenotype.

The *RanBPM^ k05201^* allele carrying a *P{lacW}* insertion in the second exon of the *RanBPM* was identified as responding poorly in the ON/OFF assay [28]. *RanBPM^ s135^* is a deletion that eliminates a maximum of 4.1 kb from the N-terminus to the middle of the gene (64747 to 68882 in AE003829), generated by excision of the element in *RanBPM^ k05201^* [29], [30]. *RanBPM^ ts7^* carries a deletion from within 10 bp of the predicted start site extending upstream toward but not including *CG12896* [8]. *RanBPM^revertant^* is a precise excision of the element in the *RanBPM^ k05201^* as determined by sequencing (data not shown). Strains used include *elav-GAL4* (Bloomington Stock Center (BSC) stock #8765), *Dmef2-GAL4* (BSC stock #27390), *tub-GAL4* (BSC stock # 5138), *247-GAL4* (Schulz et al Oncogene 12:1827 96),*386Y-GAL4* [31], *201Y-GAL4* (BSC stock # 4440), *UAS-GFP:LacZ.nls* (BSC stock # 6452) and *UAD-CD8-GFP* (BSC stock # 5130). The stock carrying the *UAS-RanBPM^ short^* construct was provided by Paul Lasko (McGill University, Canada). *The UAS-RanBPM^ long^* line was generated from the cDNA clone RH61511 (FBcl0270865, http://flybase.org/). All constructs were sequenced. *fmr1^EP3517^* (BSC, stock #6928), is a hypomorphic allele, [32] and *fmr1* ^Δ50M^ (BSC #6930) is a deletion [32].

### Behavioral Assays

#### ON/OFF

We used the ON/OFF assay to measure changes in locomotion in response to light in individual larvae as described [33], [34]. Larvae were manipulated in darkness except for a red safelight (20 W lamp with GBX-2 filter KODAK). For the assay, a single foraging third instar larva (84–90 h AEL) was placed in the center of the plate and subjected to 10 sec of light and 10 sec of dark and so forth. The light, cool white bulb, (15W, Sylvania in a Rapid Start mechanism), was controlled by a serial microcontroller (MacIO, MacBrick,) and a relay unit (AZ696) connected to a computer that ran a custom macro (NIH Image 1.62f). A Fujinon TV•Z zoom lens attached to a CCD TV camera (Elmo, TSE 272S) captured behavior. Each trial lasted 60 or 120 seconds. The semi-automated system described in detail elsewhere [6], [33], [35] was used to track and calculate the Response Index (RI) (RI =  [(path length in dark – path length in light)/total path length during the assay]) through the execution of a NIH Image macro. Alternatively, larval movement during the assay was analysed using *Dynamic Image Analysis System (DIAS)* software [36], [37]. The parameters and methodology used as previously described [34]. Briefly, centroid position over time was used to generate a larval path employed to calculate change of direction. For a qualitative analysis, perimeter stacks (larval outlines) of representative larvae were generated. All genotypes were tested for locomotion in constant dark (safe-light) as described above using the semi-automated system.

#### Feeding assays

The food intake assay was conducted as in [38] with batches of 25 larvae staged as described. Larvae were starved for two hours and placed in a drop of yeast paste laced with blue food dye for 30 min or 1.5 hours (Food Dispersal). At that the end of the assay the fraction of larvae displaying blue gut and the fraction immersed in the yeast were determined. Alternatively, larvae were placed 3.99 cm (+/−0.04, N = 7) away from the centrally- located yeast drop (Food Attraction). After 1.5 hours the fraction displaying blue gut and the fraction immersed in the yeast were determined as above. Larval behavior was captured (6 frames/min) for the duration of the assay.

#### Contact chemosensory assay

Response to NaCl was assayed essentially as described in [39] and [40]. Approximately 25 larvae were placed in the center of a petri dish divided into 4 quadrants. Opposite quadrants were filled with 1% agar in 1 M NaCl or 1% agar in water. The position of each larva was determined at 10 and 15 min intervals. Larvae that did not move outside a 1 cm radius from the original position were not counted. Similar fraction of yw, Revertants, *RanBPM* mutant and larvae remained inside the 1 cm (30–20%), while OR mostly moved out of this area by 10 min (93%).

### Statistical Analyses

We used SPSS Version 17 and The SAS System Version 9.2. The statistical tests included one-way analysis of variance (ANOVAs) and Tukey's-pairwise comparisons. In SAS we used the GENMOD Procedure with a Binomial distribution and a Link Function called ‘Logit’ to conduct a form of chi square analysis for nonparametric data. Normality tests on the residuals of the ANOVAs were conducted using the Shapiro-Wilk test. Verification of samples was performed by the F-test or Bartlett's test. The level of significance α in all tests was 0.05. All measurements are shown as mean values and SEM, and * indicates samples significantly different from control genotypes.

### Immunohistochemistry and imaging

#### CNS

Larval brains from foraging third instar larvae staged as above were treated as described [35]. Primary antibodies used: mouse anti-Elav (1∶200), anti –Repo (1∶200) and anti-FasII (1∶2) (DSHB), rabbit anti-RanBPM, (1∶1000) ([8]), rabbit anti-β-galactosidase (1∶100) (Cappel), rabbit anti-5-HT (1∶200) (Protos Biotech), rabbit anti-phosphorylated Histone3 (Upstate Biotechnologies 1∶1000) and rabbit anti-Rfamide (1∶1000) (sNPF, kindly donated by Jan Veenstra). The anti-RanBPM antibody was generated in rabbits against a N-terminal fragment of *RanBPM*. In western blotting it recognizes in wild type samples a single band of approximately 140 kD absent in samples prepared from deletion strains ([8]) Secondary antibodies used: Texas Red-conjugated goat anti-rabbit IgG (1∶200), HRP-conjugated goat anti-mouse IgG and Cy3-conjugated goat anti-rabbit IgG (1∶200) (Jackson); Alexa 594-conjugated goat anti-mouse IgG goat (1∶250), Alexa 488-conjugated goat anti-mouse IgG (1∶200) (Molecular Probes).

#### NMJ

Foraging third instar larvae were dissected in phosphate buffered saline in order to expose the musculature of the body wall, fixed at room temperature in 4% formaldehyde in PBS and incubated with goat FITC-conjugated anti-HRP 1∶500 (ICN). A Z-stack of the NMJ of muscles 6 and 7 were projected as a single image and the total number of boutons and branches counted.

#### Confocal

We used a Zeiss Axiovert 100 M and the LSM 510 image software. Brightness and contrast were adjusted using Adobe Photoshop 5.0.

## Results

### Mutations in the *RanBPM* gene cause disruption of the larval response to light and locomotion

**Figure 1:**
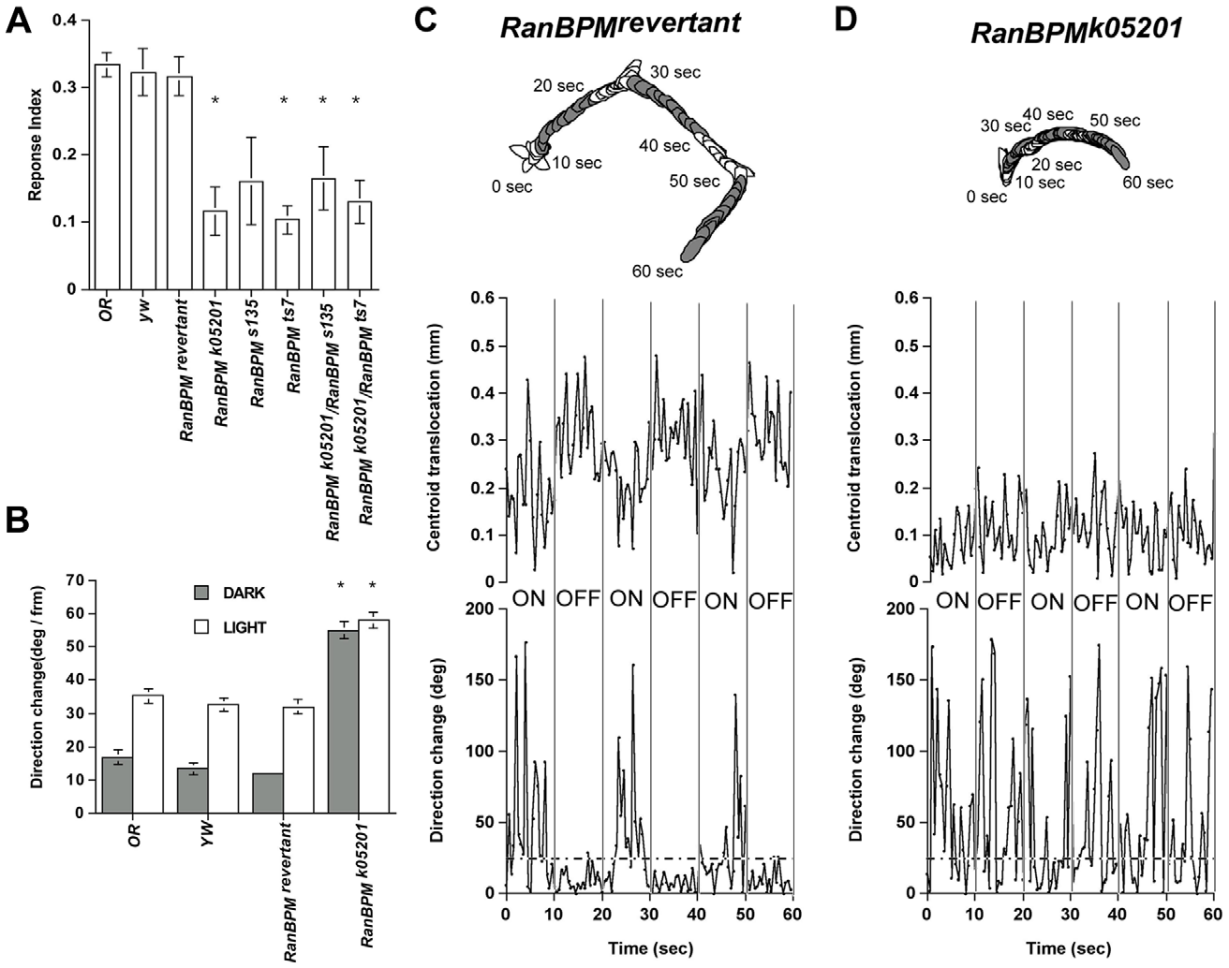
Mutations in *RanBPM* gene impair larval response to light and locomotion. Response to light is seen in the ON/OFF assay as light-induced changes to quantitative aspects of locomotion. This response was quantitated in the semi-automated system as a response index (RI =  [Distance traveled (pixels) in dark (OFF) pulses - distance traveled (pixels) in light (ON)]/Total distance traveled) (**A**). DIAS was used to calculate direction change (**B**) or response of an individual larva during the course of the assay (**C and D**). The latter is shown as empty larval outlines (perimeter stacks) depicting behavior during the light (ON) pulse, and shaded outlines behavior during the dark (OFF) pulse. Quantitative analysis of perimeter stacks are shown in the graphs below as centroid translocation (mm) and direction change (deg). Points below the 20° threshold (dashed line) indicate linear movement. Reduced *RanBPM* function disrupts the larval response to light as seen by the significant lower RI exhibited by homozygous *RanBPM^K05201^*, *RanBPM^TS7^* and heterozygous *RanBPM^K05201^/RanBPM^TS7 or^* ^S135^ (p<0.05). The response measured for homozygous *RanBPM^S135^* is markedly reduced but not statistically significant (p = 0.102) nevertheless this allele does not complement the *RanBPM^K05201^* (ANOVA F(7,131) = 7,242, p<0.0001, **A**). The control genotypes (OR, *yw* and *RanBPM^revertant^*) show the increased change of direction triggered by light, that characterizes the photophobic behavior of the foraging larva (**B** and **C**). In contrast, in the *RanBPM^K05201^* mutants not only is locomotion markedly reduced but it is also uncoordinated as seen by the significant higher change of direction that occurs during the dark and light pulses (**B** p<0.001 and **D**), (ANOVAs for direction change during light F_(3,30)_ = 5.934, p<0.03 and during dark F_(3,30)_ = 25.351, p<0.0001, **B**). In all genotypes N≥12.

The third instar larva light avoidance response is characterized by interruption of forward peristalsis, vigorous head-swinging followed by direction change, leading to a reduction in locomotion ([6], [34] and Figure 1C). Light avoidance can be measured in the ON/OFF assay, as changes to different aspects of locomotion of a single larva exposed to intermittent pulses of light [33], [34].

Larvae homozygous for a P-element insertion in the predicted second exon of the *RanBPM* gene [8], henceforth named *RanBPM^k05201^* or for alleles generated by imprecise excision of this element, displayed a response index in the ON/OFF assay (RI =  [(path length in dark – path length in light)/total path length during the assay]) which was significantly lower than that of controls (Figure 1A). DIAS-assisted analysis of larval locomotion during the assay revealed that in these mutants locomotion is reduced and uncoordinated as seen by the equally high degree of direction change during the lights on and off pulses (Figure 1B, C and D). *RanBPM^K05201^* mutant larvae also displayed reduced locomotion, in constant dark (Figure S1).

The *RanBPM* gene was previously characterized as a vital gene (Table S1) playing a role in ovary development [8]. A deletion of the upstream region, (*RanBPM^ts7^*) generated by these authors was used in behavioral assays as described above. Our results suggest that *RanBPM^ts7^* allele represents a less severe disruption of the *RanBPM* gene function. While larvae homozygous for the *RanBPM^TS7^* allele show a reduction in the response to light similar to that exhibited by larvae carrying the *RanBPM^k05201^* allele (Figure 1A), these mutants move more vigourously than the others (143.88 pixels +/−12.41 versus 26+/−3.96 for *RanBPM^k05201^* N>15). We conclude that *RanBPM* gene function is required for larval response to light and for coordinated locomotion.

### *RanBPM* function is required for larval feeding behavior

Larvae homozygous for either the *RanBPM^k05201^* or *RanBPM^s135^* alleles, but not for the *RanBPM^ts7^* allele, were smaller than *yw* control specimens at chronologically the same stage (Figure S2 and data not shown) This size difference was apparent from the early third instar stage but not before.

Reduced larval growth can occur as a consequence of decreased food intake, arrest of cellular growth or a decrease in endoreplication [3], [41], [42], [43], [44]. In order to address whether insufficient food intake is associated with the reduced size of *RanBPM* mutants, we conducted feeding assays essentially as described previously [38]. Following a 2-hour starvation period, early foraging third instar *RanBPM* mutants were placed on yeast paste containing food dye for 30 min. While circa 88% of the control larvae ingested food within 30 minutes of being placed on the yeast paste, only 14–30% of *RanBPM* mutants (*RanBPM^k05201^* and *RanBPM^s135^* homozygous or heteroallelic combinations) displayed the characteristic blue gut (Table 1 and Table S2). As expected, given its normal size, mutant larvae homozygous for the *RanBPM^ts7^* allele ate like the control genotypes (Table S2).

**Table 1:** Feeding Rescue.

	Yw	RanBPM^k05201^	RanBPM^k05201^;UAS-RanBPM	
			Long	Short
*yw*	88.667±0.882			
*RanBPM^k05201^*		29.67±4.26	19.52±1.62	25.6±1.89
*RanBPM^k05201^;elav-GAL4*		33±2.73	83±2.65	86.33±3.71
*RanBPM^k05201^;247- GAL4*		27±3.16	64±2.65	81±2.31
*RanBPM^k05201^;386-GAL4*		24.7±4.12	35±4.16	85±3.06
*RanBPM^k05201^;Dmef2-GAL4*		37±4.09	83.33±2.6	71.67±1.45

*RanBPM^k05201^* homozygous mutants had a significant reduction in presence of blue guts compared to wild type YW (X^2^ (15) = 426.63 p<0.0001). *RanBPM^k05201^* homozygous mutants, those carrying a *GAL4* driver alone or those carrying *UAS –RanBPM* short or long alone were not significantly different from one another. *elav-GAL4* (p<0.0001), *MB247-Gal4* (p<0.0001), *Dmef2-Gal4* (p<0.0001), were able to rescue the feeding phenotype with both *RanBPM* short and long isoforms. *386-GAL4* was able to rescue the feeding phenotype with the short isoform (p<0.0001) but not the long (p = 0.0719).

**Figure 2:**
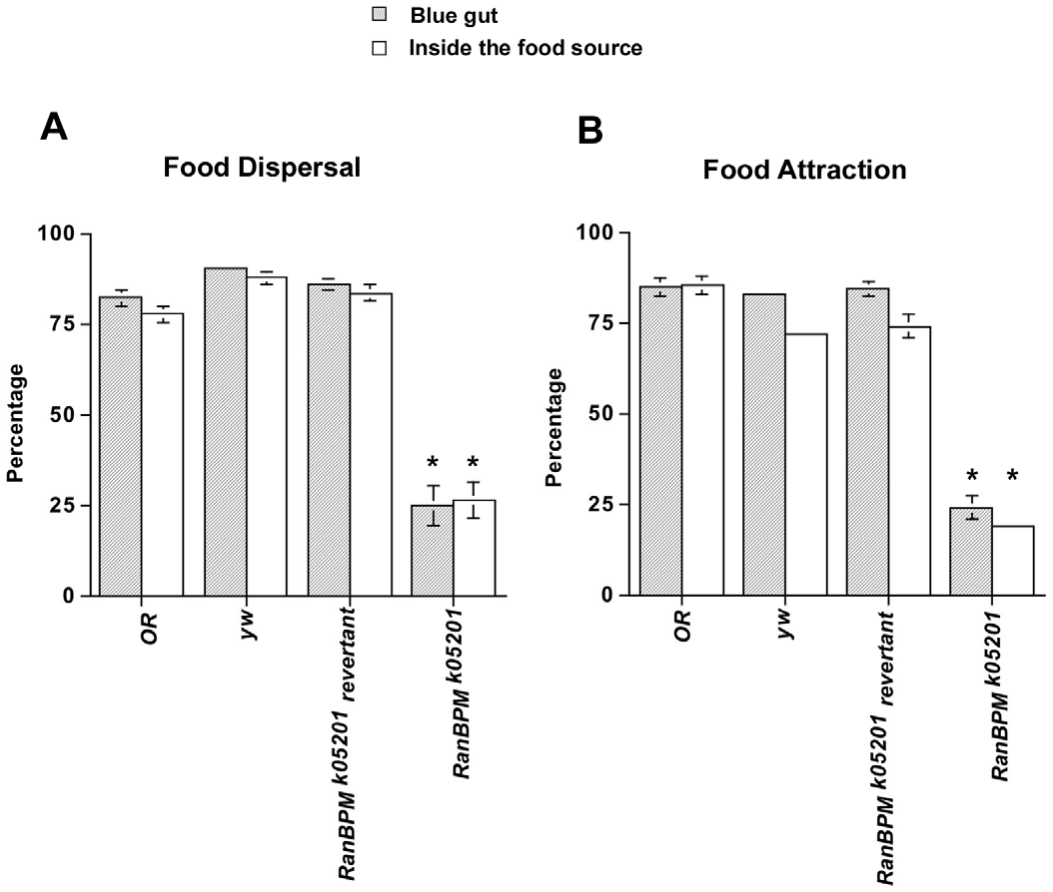
*RanBPM^K05201^* mutants feed less and are not attracted to food. Batches of 25 3^rd^ instar foraging larvae were starved for 2 hours and then placed inside (**A, Food Dispersal**) or outside the food (**B, Food Attraction**) laced with blue dye for 1.5 hours. The proportion of larvae that were in the food was determined at the end of the assay. All larvae were examined for the presence of blue matter in the gut indicative of food intake. *RanBPM^k05201^*mutants when placed inside the food in the beginning of the assay ingest food significantly less than control larvae (**A** shaded bars, x^2^ = 94.68, DF = 3, p = 0.0001) and are found immersed in the food in a significantly smaller number than control (**A** empty bars, x^2^ = 83.31 DF = 3, p = 0001). Similarly, when placed outside the food plug *RanBPM^k05201^* mutants feed significantly less (**B** shaded bars, x^2^ = 120.89, DF = 3,) and are attracted to the food less (**B** empty bars x^2^ = 121.09 DF = 3) when compared to control larvae. In all experiments * p<0.0001, N>100.

To further investigate the feeding phenotype of *RanBPM* mutants, we allowed larvae to feed for a longer period (1.5 hours) The fraction remaining in the food and the fraction displaying a blue gut were determined as above. Control larvae, vigorously ingested food and remained for the most part immersed in the yeast for the duration of the assay. In contrast, a large fraction of *RanBPM* mutant larvae left the yeast drop and were found at the end of the assay outside the food source (Figure 2A).

In order to determine whether the *RanBPM* mutants were attracted to yeast we placed the larvae outside the centrally located yeast plug. After 1.5 hours the fraction that moved into and remained in the food and the fraction displaying a blue gut was determined as above (Figure 2B). Larval behaviour during the assay was captured and a movie of a representative assay is available as supplementary material (Movies S1 and S2).

As expected, at the end of the assay, a large fraction of the control larvae were found immersed in the food and displayed the characteristic blue gut of fed larvae. In contrast, a large fraction of the *RanBPM* mutants did not move into the food source or did not eat (Figure 2B). The behavior of *RanBPM* mutants captured during the assay showed that these larvae moved around the plate and came close to the yeast drop but rarely entered and/or remained immersed in it (Movie S2). Interestingly, lack of *RanBPM* function does not impair the larva's repulsion to high concentrations of NaCl (Figure S3).

The apparent lack of interest in food displayed by *RanBPM* mutant larvae is reminiscent of precocious wandering behavior typical of the older third instar larvae, suggesting that lack of *RanBPM* function disrupts processes that underlie food intake. The multiple phenotypes displayed by *RanBPM* mutants suggest that this is a pleiotropic gene required in a variety of tissues. Alternatively, these phenotypes may be due to lack of gene function in a defined group of cells.

### *RanBPM* mutations cause reduction in cell proliferation but no apparent disruption in differentiation of identified larval neurons

**Figure 3:**
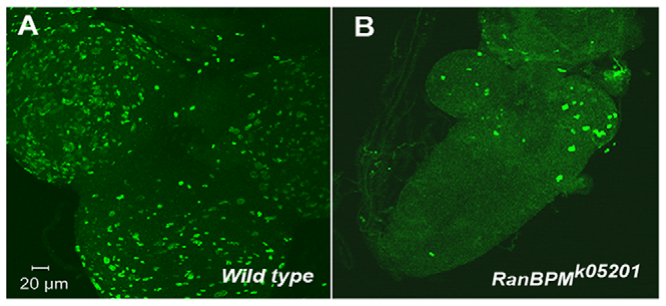
*RanBPM* function is required for cell proliferation. Confocal micrographs of larval brains immunolabeled with anti-phosphoH3, detected with Alexa 488 secondary antibody. Images shown are projected Z-stacks of 10–13 (wild type) or 5–7 sections (*RanBPM^K05201^*) sections at 2 µm intervals. The *RanBPM^K05201^* mutant CNS (**A**) is smaller than the wild type control (**B**) and show reduced phosphoH3 labeling, indicating that lack of *RanBPM* gene function disrupts proliferation.

We asked whether reduced larval growth was associated with reduced cell proliferation as seen by phosphorylated Histone3 (phosphoH3) immunolabelling of whole-mount *RanBPM* mutant larval brains. Indeed, smaller than wild-type brains of larvae homozygous for *RanBPM^k05201^* showed a dramatic reduction in phosphoH3 immunolabelling (Figure 3). Surprisingly, we did not detect major morphological defects in these mutant larvae. Labeling of dissected larval brains with a number of different neuronal markers such as 5-HT [45], FasII [46], sNPF [47], [48], *386-GAL4* [31], and *247-GAL4* [49], [50], NPF [51] indicated that *RanBPM* is not required for the differentiation and/or maintenance of larval neurons (Figure S4 and data not shown). The smaller size of the CNS is reflected in the apparent smaller volume of the MB neuropil (Figure S4 A, A'). Moreover, we detected a small but statistically significant reduction in the number of serotonergic cell bodies with no apparent disruption in the pattern of projection of these neurons (Figure S4 H–J).

### The long isoform of *RanBPM* is highly expressed in the Kenyon neurons of the mushroom body

The *RanBPM* gene encodes two protein isoforms with predicted masses of 67 and 140 kD (named RanBPM^short^ RanBPM^long^). Both proteins contain the SPRY domain, LisH motif, CTLH motif and the CRA domain. The long isoform differs by the presence on the N terminus of a non-conserved glutamine rich segment [8].

**Figure 4:**
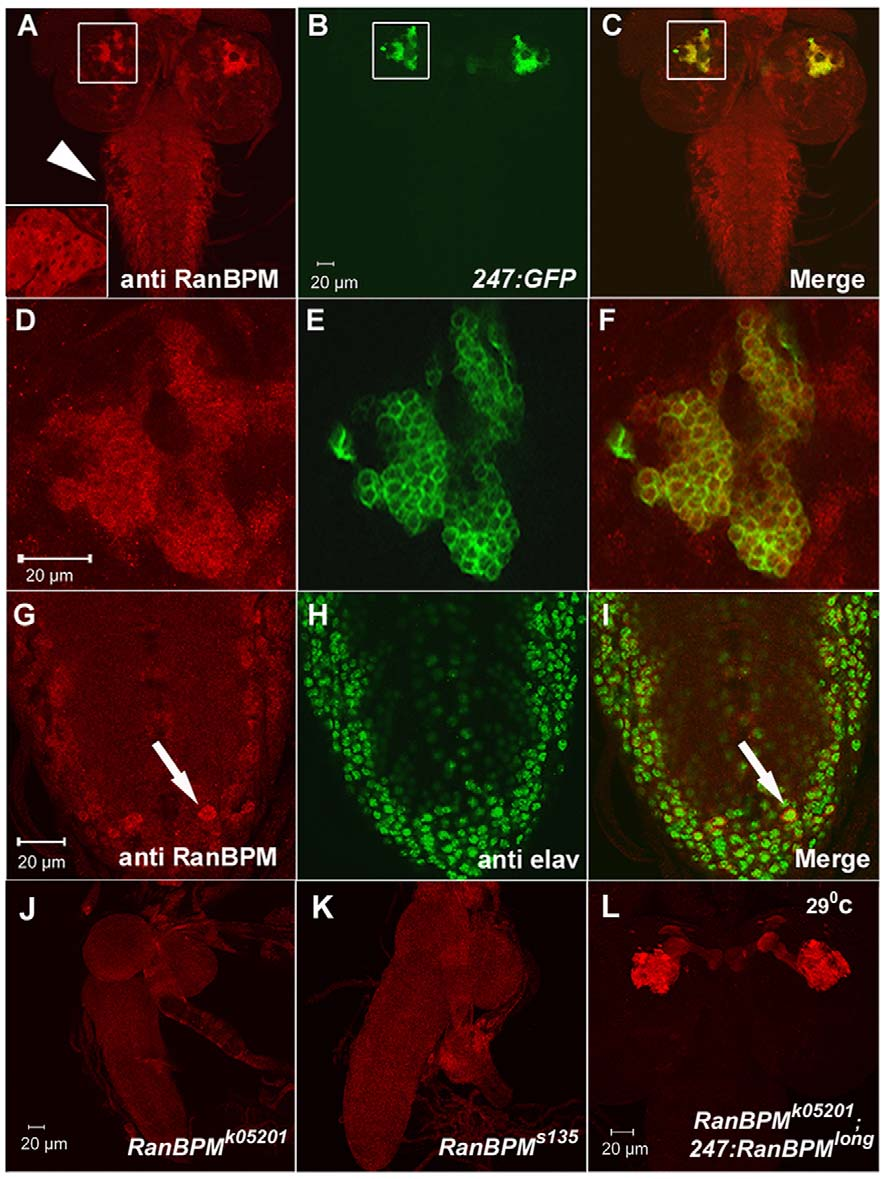
RanBPM^long^ is highly expressed in the Kenyon cells of the mushroom body. Confocal micrographs of wild type larval CNSs immunolabeled with anti-*RanBPM^long^* detected by Cy3-conjugated secondary antibody (red) and carrying a MB reporter construct *247-GAL4/UAS-CD8-GFP* (green, *247:GFP*, **A–F**) or double labeled with a pan neuronal antibody (anti-elav, green, **G–I**). RanBPM labeling is found in the ventral cord (arrowhead in **A**), the ring glands (inset in **A**) and in two bilateral clusters of neurons located in the brain hemispheres (box in **A–C**). Boxed area in **A–C** is magnified in **D–F**. These neurons are the Kenyon cells of the MB as seen by extensive co-localization with the MB reporter *247-GFP*. In the abdominal portion of the ventral cord, *RanBPM* expression is found widely but not at the same level in all cell bodies (arrow in **G** and **I**). CNS dissected from homozygous mutant larvae (**J**, *RanBPM^K05201^*; **K**, *RanBPM^S135^*), and the *RanBPM^K05201^* mutant expressing the long isoform under the regulation of the MB specific driver *247-GAL4* (**L**, *RanBPM^K05201^; 247:RanBPM^long^*) were labeled with anti-RanBPM antibody (red) that recognizes the long isoform. The CNSs of larvae homozygous for the two most severe alleles show reduction in the volume of the CNS and absence of the characteristic RanBPM expression pattern in the brain (**J and K**). In the rescued sample (**L**, *RanBPM^K05201^; 247:RanBPM^long^*), RanBPM immunolabelling is restricted to the MB cell bodies and neuropil, the latter represents ectopic expression due to the high level of expression of this *GAL4* driver (compare **A** with **L**).

To investigate the expression of the *RanBPM* gene we used an antibody directed against the N-terminus unique to the 105 kDa isoform kindly provided by Paul Lasko (McGill University,[8]). In the brain lobes and ventral nerve cord (VNC), *RanBPM* was expressed widely but not in all neurons labeled by the pan-neural marker *elav* [*52]*, [53]. No staining was observed in the proliferating centers of the optic lobe or in the photoreceptors (Figure 4A). Consistent with this observation, no co-localization was detected with phosphoH3 immuno-labeling (Figure S5A–F). Similarly, *RanBPM* immuno-labeling did not co-localize with that of the glial marker, *repo* (Figure S5G–L), [54]. In the CNS, *RanBPM* labeling appeared to be cytoplasmic but not restricted to the neuronal cell bodies (e.g. Figure 4G–I). We did not detect *RanBPM* labeling in the neuromuscular junction (NMJ) [28]. Some, but not all motoneurons labeled by the motoneuron reporter D42-Gal4 [55], also expressed *RanBPM* (data not shown).

In the brain hemispheres *RanBPM* was highly, but not exclusively expressed in a bilateral dorsal cluster of neurons. This labeling co-localized with the expression of a mushroom body marker (*247-GAL4;UAS-CD8-GFP,* [*50*]; Figure 4A–F). At this level of resolution it appeared that all *RanBPM*-expressing cells present in this dorsal cluster co-expressed the mushroom body-specific element *247-GAL4*. While we did not detect *RanBPM* expression in the imaginal discs, we did find expression in the muscle fibers attached to the mouth hooks and in the cytoplasm of the ring gland (data not shown and insert in Figure 4A). This pattern of expression was not detected in *RanBPM* mutant larvae, demonstrating that it is not due to cross reactivity of this antibody (Figure 4J and K). Moreover, targeted expression of the long isoform in the mutant background restored *RanBPM* expression, as detected by immunolabelling with this antibody, in a pattern consistent with that of the *GAL4* driver employed (Figure 4D–E). No signal was detected in wild type larval brain specimens when we used an antibody generated against a portion of the *RanBPM* open reading frame common to both proteins (kindly provided by Paul Lasko, McGill University [8]). Therefore, we do not know whether in the larval CNS expression of the short isoform differs from that of the long isoform. Nevertheless, as described below, targeted rescue experiments indicate that these two isoforms are functionally equivalent.

### Nervous system expression of either *RanBPM* isoforms rescues all *RanBPM* larval behavioral phenotypes

In order to identify the cells or group(s) of cells in which *RanBPM* expression plays a role in larval behavior, we generated *RanBPM* constructs whose expression can be targeted to particular cells and tissues under the control of tissue-specific *GAL4* transgenic constructs [56]. Using standard genetic crosses we created homozygous *RanBPM^k05201^*mutant flies carrying the *RanBPM^long^* or *RanBPM^short^* cDNA under the control of the *GAL4*-responsive DNA binding site (*UAS*) as well as different *GAL4* drivers.

Expression of either *RanBPM* isoform under the control of the *tub-GAL4* driver reduced the recessive lethality associated with *RanBPM* mutations from 100% to 61–69% (N>200). demonstrating that the both constructs contained all the information required to restore *RanBPM* gene function and that the two isoforms are functionally equivalent.

**Figure 5:**
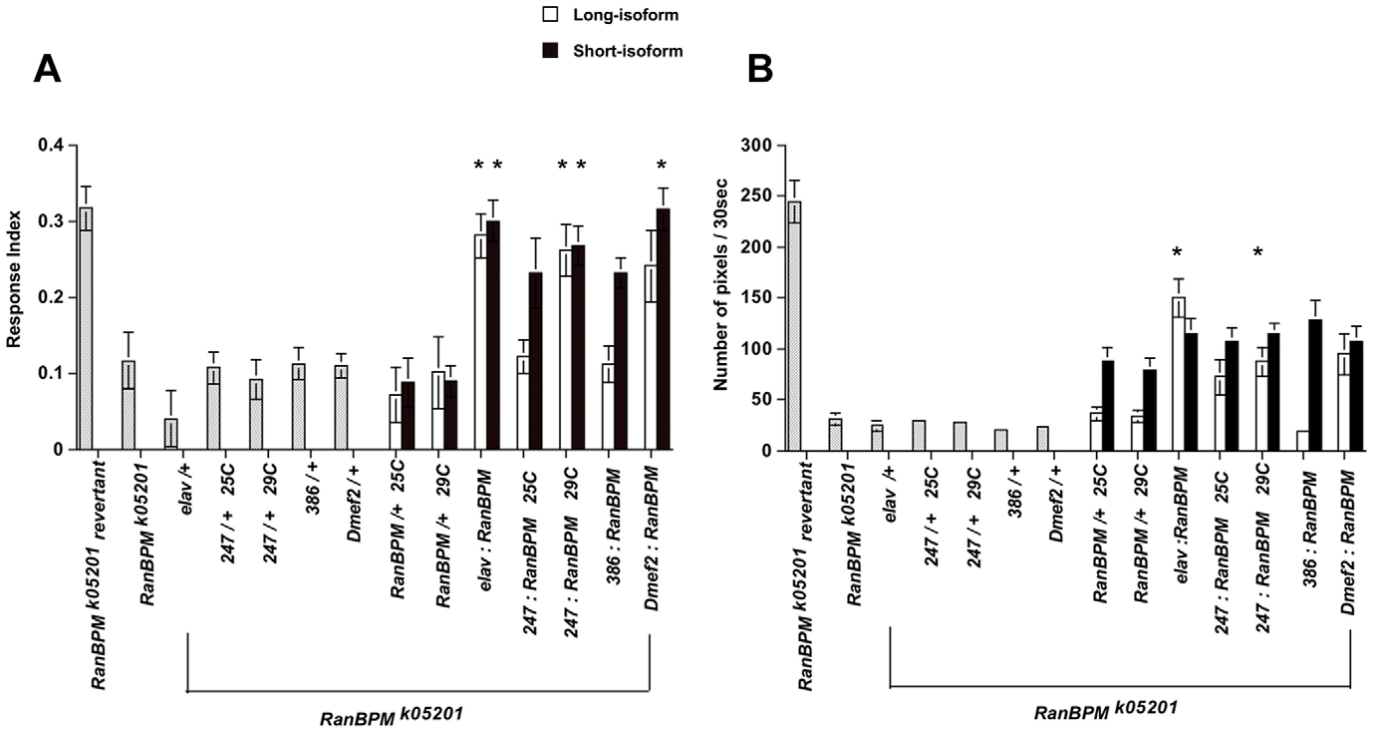
Targeted expression of *RanBPM* in subsets of neurons rescues the control of locomotion by light and the locomotion phenotypes of *RanBPM^k05201^* mutants. **A-** The presence of any of the *GAL4* drivers or either one of the UAS constructs did not significantly change the larval response to light. Pan-neural (*elav-GAL4*) expression of either *RanBPM^long^* or *RanBPM^short^* constructs significantly increased the response to light of *RanBPM^k05201^* mutants relative to that of *RanBPM^k05201^* mutants alone or carrying one copy of either one the *GAL4* drivers or UAS constructs. Expression of the short *RanBPM* isoform under the control of *247-GAL4* (29°C) and *Dmef2-GAL4*, significantly increased the response to light phenotype of *RanBPM^k05201^* mutants. *RanBPM* mutant larvae expressing the *UAS-RanBPM^long^* construct in the MB under the control of the *247-GAL4* driver displayed significantly higher response to light only when grown at 29°C. * p<0.05 N≥10 (ANOVA: F_(10.082)_ = 20,256, p<0.001). **B**-Locomotion of *RanBPM^k05201^* mutant for *RanBPM^K05201^* mutants expressing the long or short isoform under the control of various *GAL4* drivers is shown as the number of pixels (mean ± SEM) in 30 sec of the assay. Locomotion was significantly reduced in *RanBPM^k05201^* mutant larvae relative to revertant control and did not improve significantly when these mutants carried one copy of any of the GAL4 drivers or one copy of the long isoform construct. Expression of the long RanBPM isoform under the control of *elav-Gal4 and 247-GAL4* (29°C) significantly increased locomotion above that of all mutant controls to levels still below that of the *RanBPM^K05201^* revertant control. Presence of the short RanBPM isoform construct significantly increased the locomotion of *RanBPM^K05201^* mutant larvae and that was not increased when a GAL4 driver was introduced. *p<0.05, N≥11 (ANOVA: F_(19.491)_ = 20,257, p<0.001).

Next, we asked whether expression in the nervous system was sufficient to rescue *RanBPM* phenotypes. Targeted expression of either the short or long *RanBPM* isoform using the pan-neural driver ***e*** *mbryonic* ***l*** *ethal* ***a*** *bnormal* ***v*** *isual system (elav)-GAL4* effectively rescued the response to light phenotype of *RanBPM* mutants (Figure 5A) as well as the feeding phenotype (Table 1). Using the *elav-GAL4* driver overall locomotion of *RanBPM* mutant larvae markedly improved but was only significantly above background when the long isoform construct was used. (Figure 5B). Interestingly lethality was partially rescued as seen by the survival of a small fraction (∼14%) of *RanBPM^k05201^* mutants flies to adulthood (Supplementary Table S1 and S3). These flies displayed a spread wing phenotype similar to that of *Dichaete* (*D*) mutants also seen in all escapers of heteroallelic combinations that supported survival to adulthood.

The *elav* gene and the *elav-GAL4* line used in this study are highly expressed in postmitotic neurons of the central and peripheral nervous system [52] and transiently expressed in glia and neuroblasts [53]. Therefore, it is possible that expression in cells other than differentiated neurons contributed to the observed rescue of the mutant phenotypes. Targeted expression of either *RanBPM* isoform using the glia-specific driver *repo-GAL4* did not rescue any of the phenotypes (data not shown) and co-localization with the anti-Repo antibody was not observed (Figure S5 G-L). However, the inability to rescue a given mutant phenotype when using a heterologous promoter may be due to target gene expression that is below the threshold required, rather that inappropriate tissue-specific expression. Taken together, our results demonstrate a role in larval behavior, for *RanBPM* gene function in the nervous system, perhaps in postmitotic neurons, and suggest that the lack of *RanBPM* gene function in this tissue contributes to the observed lethality.

### Expression of *RanBPM* in the Kenyon neurons of the MB is sufficient to rescue the response to light and feeding phenotypes

Given that the long isoform of *RanBPM* is highly expressed in the MB neurons, we asked whether expression in these neurons was sufficient to rescue *RanBPM* mutant phenotypes. To that end, we used *GAL4* drivers whose expression overlapped in the MB. *247-GAL4* is derived from an enhancer found in the *Dmef2* promoter and is highly and nearly exclusively expressed in the MB neurons throughout larval development until adulthood ([50] and data not shown). *Dmef2-GAL4* is expressed in several neurons, in addition to those of the MB, as well as in somatic muscles [49]. The pattern of expression of *386-GAL4* extends to numerous peptidergic neurons [31] and includes MB neurons (Figure S4 C-D').

The performance of *RanBPM^k05201^* mutants expressing either isoform under the regulation of MB *GAL4* drivers in the ON/OFF assay, is shown in Figure 5 panel A. Targeted expression of at least one of the two *RanBPM* isoforms under the regulation of two (*247* and *Dmef2*) of the three MB *GAL4* drivers was sufficient to restore the response index of *RanBPM* mutants to levels that are not statistically different from that of control strains. Targeted expression of the long isoform using the *386-GAL4* driver markedly improved the response of mutants but this was not statistically significant. In the case of the *247-GAL4* driver, marked and significant rescue occurred using either isoform when larvae were grown at 29°C, the optimal temperature for the function of the yeast transcription factor *GAL4*

In rescued larvae (*RanBPM^k05201^*; *247:RanBPM^long^*) expression of the long isoform of *RanBPM* is restored in the MB neurons (Figure 4L). The subcellular localization within the MB structure however, differs from that of wild type samples. In the rescued specimens *RanBPM* immunolabelling is found in the cell bodies, the calyx and in the pedunculi, while in wild type specimens it is restricted to the cell bodies (Figure 4 compare A to L). This is likely due to the heterologous promoter, in this case the *247-GAL4* driver, driving the expression of the *UAS-RanBPM* target construct to levels above that of wild type, thereby causing the ectopic expression of this protein in the pedunculi.

Significant improvement of locomotion occurred in mutants expressing the long isoform in the MB neurons when grown at 29°C (*RanBPM^k05201^*; *247:RanBPM^long^*, Figure 5B). While marked increased in locomotion was also observed when the other MB *GAL4* drivers were used, they were not statistically significant. Lethality was marginally rescued by expression of *RanBPM^long^* under the control of *Dmef2-GAL4* and not at all when *247-GAL4* and *386-GAL4* were employed (Table S1 and S3). In contrast feeding was completely restored when all three MB *GAL4* drivers were employed (Table 1). We concluded that *RanBPM* expression in the Kenyon neurons is sufficient to rescue the behavioral phenotypes of *RanBPM* mutants.

### *RanBPM* gene function contributes to FMRP-dependent processes

*RanBPM* was previously identified in a yeast two-hybrid screen as a putative partner of FMRP [18]. FMRP is an RNA binding protein implicated in the transport, translational control and metabolism of mRNAs encoding proteins involved in synaptic plasticity (reviewed by [23], [57], [58]. The *Drosophila* orthologue, *dfmr1*, has been used extensively as a model for FMRP function in the regulation of synaptic structure and function (reviewed by [59]). *dfmr1* mutants display over -elaboration of neuronal structure exemplified by midline crossing of intrinsic MB neurons as well as increased branching and bouton number at the larval neuromuscular junction (NMJ)[20], [21], [32], [60]. Moreover *dfmr1* function is required for activity dependent pruning of MB axons [24].

**Figure 6:**
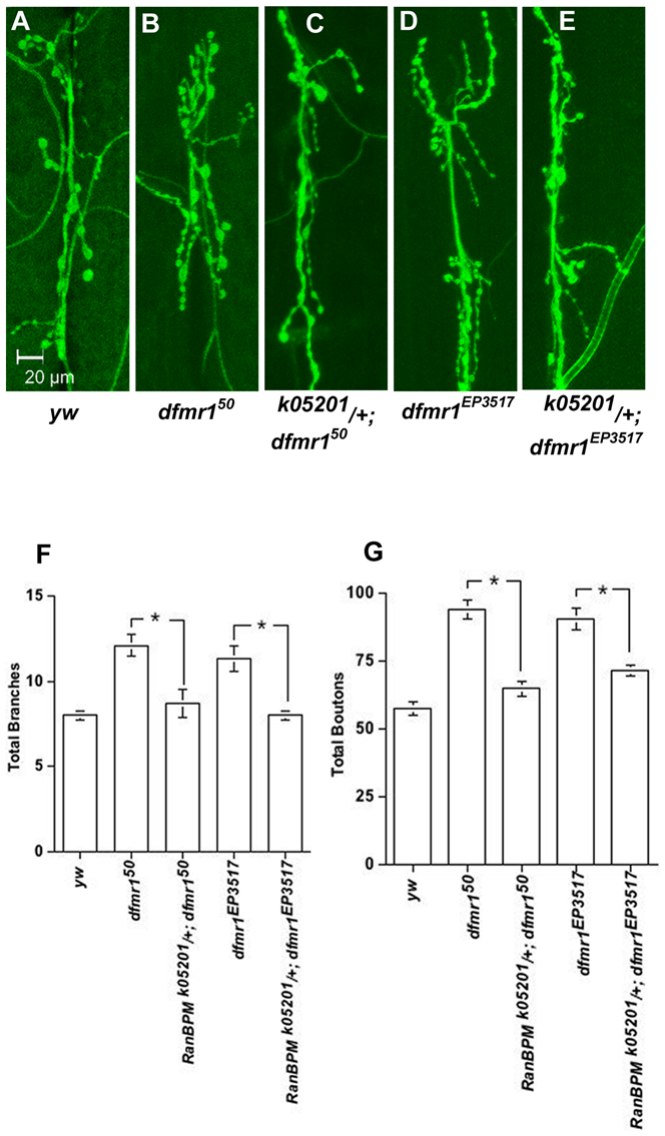
*RanBPM* function is required for FMRP-dependent development of larval NMJs. Confocal micrographs of larval NMJs (muscles 6 and 7) labeled with FITC-conjugated anti-HRP (**A–E**), show the overgrowth phenotype characteristic of *dmfr1^50^* (**B**) and *dmfr1^EP3517^* (**D**). Introduction of one copy of the lack-of-function allele *RanBPM^K05201^* partially suppresses this phenotype (compare **B** to **C** and **D** to **E**). These observations are supported by quantitative analysis, in which total number of branches and boutons are counted (**F, G**). Reduction of *RanBPM* function significantly reduces the number of branches (**F**, ANOVA: F_(4,42)_ = 9.713 p<0.001) and boutons (**G**, ANOVA: F_(F4,41)_ = 28.382 p<0.001) of both *dfmr* mutants (N≥8).

We asked whether *RanBPM* function is required for FMRP-dependent processes. To that end, we conducted genetic epistasis analysis to determine whether *dfmr1* mutant phenotypes are modified (enhanced or suppressed) by changes in the level of *RanBPM* function. *RanBPM^k05201^* mutations cause lethality during late larval development while the *dfmr1* mutant alleles used here, *dfmr1^EP3517^*, *dfmr1^Δ50M^* cause lethality at the pharate adult stage and are hypomorphic and amorphic mutations respectively. Interestingly *RanBPM;dfmr1* double homozygotes die during embryogenesis. We asked whether reduction in RanBPM function modifies the NMJ overgrowth phenotype of *dfmr1* mutants. *dfmr1* mutant NMJ is characterized by pronounced synaptic overgrowth as seen by nearly 50% increase in branching and in the total number of boutons ([32], [60]; and Figure 6). While the NMJ of double mutants does not appear entirely normal, introduction of one copy of the lack of function allele *RanBPM^k05201^* is sufficient to suppress the overgrowth NMJ phenotype. Increased branching and bouton number is reduced in these double mutants to levels comparable to that of wild type controls. These observations indicate that *RanBPM* function is required for the expression of *dfmr1*phenotype and suggest that *RanBPM* and *dfmr1*function converge in the mechanisms underlying synaptic growth.

## Discussion

The *RanBPM* gene was first identified in vertebrates and proposed to function as a scaffolding protein (reviewed in [61]). *RanBPM* mutations in vertebrate model systems are not yet available. Therefore, its precise role in these processes remains to be established. The first reported mutational approach to *RanBPM* function was in *Drosophila*. In this model organism, *RanBPM* was shown to regulate the development of the female germ line niche [8].

We report a novel role for *RanBPM* in larval behavior. *RanBPM* mutant larvae showed normal growth and behavior until the late second instar stage. Soon after the last molt, growth and food intake ceased. Our results suggest that this is due to suppression of the food seeking behavior characteristic of this stage. Mutant larvae moved away from the food, displaying precocious wandering, a hallmark behavior of the late third instar stage (Figure 2 and Movie S2). Moreover, light-induced changes to locomotion were nearly abolished, and these larvae displayed sluggish and uncoordinated locomotion (Figure 1).

Consistent with the high level expression of *RanBPM* in the MB neurons we found that targeted expression in these cells was sufficient to restore light-induced changes in locomotion and the feeding phenotypes to nearly wild type levels (Figure 5 and Table 1). Lethality was partially rescued when the *elav-GAL4* or the *Dmef2-GAL4* driver were used (Table 1). The latter (*Dmef2-GAL4* driver) is expressed in CNS neurons that include MB Kenyon cells as well as somatic muscles. Rescue of lethality was not achieved when the *247-GAL4* driver, a selective MB marker, was used (Table S3). Therefore, it is not clear whether the suppression of feeding phenotype is the sole cause for the recessive lethality caused in *RanBPM* mutations.

Rhythmic behaviors such as larval locomotion, are generated by neuronal networks called central pattern generators (CPG). Sensory input provided by the multidendritic sensory (MD) neurons is essential to coordinate the rhythmic peristalsis that constitutes the larval forward movement [62], [63]. Therefore, the current model for the coordination of larval movement demands the proper development and synchronized function of four groups of cells: neurons that constitute the CPG, MD neurons, motoneurons and body wall muscles. The uncoordinated phenotype of *RanBPM* mutants may be due to lack of gene function in any of these cell types. Collectively, the GAL4 drivers employed in our studies are expressed in all of these different cell types. However while CNS expression, in particular in the MB, neurons was sufficient to restore the ability of *RanBPM* mutant larvae to display the characteristic light-induced changes in locomotion, none restored locomotion to levels similar to that of control strains. It is possible that the GAL4 drivers employed did not provide an adequate level and/or timing of *RanBPM* expression that reached the threshold required. Additional experiments are required in order to explore the role of *RanBPM* function in the various cellular components that contribute to this rhythmic behavior and in particular the function of MB function in the control of larval locomotion.

Extensive evidence has accumulated pointing to the MB, in adult flies, as the site of olfactory learning [64]. Additional roles for MB neurons in adult behavior include regulation of motor activity, centrophobism, habit formation and saliency-based decision-making [65], [66], [67], [68]. In the *Drosophila* larvae the role of MB neurons has not been as extensively investigated.

MB function has not been directly implicated in *Drosophila* feeding behaviour. Interestingly the *Drosophila* peptide sNPF, which is structurally related to the mammalian neuropeptide Y (NPY) a regulator of food consumption, is expressed in larvae and adults, in a large subset of MB intrinsic neurons and in neurons located in the ventral cord [47], [48], [69]. Ubiquitous expression of dsRNA constructs targeting sNPF mRNA reduced food intake in *Drosophila* larvae and adults [69]. Whether sNPF expression in MB neurons is implicated in this phenotype is yet to be established. Expression of sNPF in *RanBPM* mutants appeared normal at the level of resolution afforded by immunolabelling and confocal microscopy. Given the large number of cells labeled by sNPF antibody a clonal approach must be used in order to evaluate the projection of individual neurons in a *RanBPM* mutant background.

We found that while the size of *RanBPM* mutant larvae is reduced, cell fate and differentiation appeared normal (Figures S2 and S4). Smaller larval size is reflected in the nervous system, as a marked reduction in the number of actively dividing cells. Interestingly, in *RanBPM* mutants, mitotically-active cells are nearly absent in the abdominal portion of the ventral cord while more anteriorly, in the thoracic portion and brain hemispheres, proliferation is markedly reduced but not absent (Figure 3). This phenotype is similar to that reported for newly-hatched larvae allowed to feed for 1 day and kept in amino acid-free medium for three days thereafter. It reflects the anterior to posterior wave of re-entry of neuroblasts into the cell cycle triggered by the first feeding of the newly-hatched *Drosophila* larva [70].

Impaired cell proliferation in *RanBPM* mutants is a non-autonomous phenotype. *RanBPM* expression in the nervous system was not detected in mitotically-active cells. Targeted expression of *RanBPM* in a subset of the CNS neurons rescued the reduced larval size and reduced CNS proliferation phenotypes. These observations are consistent with the notion that, in *RanBPM* mutants, reduced size and cell proliferation may be due to precocious cessation of foraging and food ingestion with consequent starvation. Starvation in turn, leads to the reduction of cell proliferation in the nervous system and reduced larval size due to inhibition of endoreplication and larval cell growth.

*pumpless* (*ppl*) and *klumpfuss* (*klu*) mutants display suppression of food-seeking behavior and reduced size similar to that observed in *RanBPM* mutants but move vigorously and coordinately suggesting that disruption of locomotion detected in *RanBPM* mutants is not necessarily due to lack of food intake [3], [42]. *ppl* encodes a subunit of the amino acid glycine cleavage system and is highly expressed in the fat body [42]. In contrast *klu*, encodes zinc finger containing transcription factor, which is widely expressed in the nervous system and is involved in the regulation of proneural proteins [3], [71].

The current model postulates the existence of humoral mitogenic signal(s) secreted by the fat body and regulated by amino acid ingestion, which may also regulate larval feeding behavior [42], [70]. Amino acid ingestion triggers the secretion, by the fat body, of the *Drosophila* Acid-Labile Subunit (dALS). Binding of dALS to *Drosophila* Insulin-Like Peptides (DILPs) in turn regulates their bioavailability [72]. Hyperactivation of insulin receptor/phosphoinosite 3-kinase (Inr/P13K) signaling in the fat body induces larval wandering –like behavior as seen by a food dispersal phenotype [73], reviewed by [74] and[75]. The focus of the suppression of larval feeding and reduced cell proliferation phenotype in *RanBPM* mutants is in the nervous system. Therefore it is not unreasonable to suggest that *RanBPM* mutants may be deficient in the reception and/or transduction of humoral signals, perhaps derived from the fat body, that trigger and sustain larval foraging behavior in order to ensure adequate food intake. The finding that feeding behaviour is rescued in *RanBPM* mutants in which *RanBPM* function is selectively restored to the MB neurons suggest that this structure plays a role in feeding behaviour (Table 1).

Several studies report direct binding of *RanBPM* to a number of different proteins (e.g. [11], [12], [13], [14], [15], [16], [17], [76], [77] reviewed by [61]). However the functional relevance of several of these interactions is yet to be established. One of the potential partners of *RanBPM* function, as established in protein binding assays, is the RNA binding protein FMRP [18]. Lack of FMRP function is the underlying cause of the more prevalent form of inherited mental retardation [19]. FMRP function is highly conserved and is required for mRNA transport and translational suppression in the context of synaptic plasticity. In *Drosophila* as well as in other model systems, lack of *dfmr1* function promotes synaptic elaboration[20], [21], [22], [32].

As a first step toward the identification of signaling pathways or biochemical functions impacted by *RanBPM* loss, we carried out a genetic epistasis experiment between *RanBPM* mutation and mutations of the Drosophila orthologue of FMRP, the *dfmr1* gene. The finding that reduction in *RanBPM* function suppresses the NMJ overgrowth phenotype of *dfmr1* mutants suggest that RanBPM contributes to FMRP dependent processes (Figure 6).

Our experiments did not address whether this is a direct or indirect contribution. It is possible that in the context of NMJ development, *RanBPM* functions as a component of a protein complex, that positively regulates *dfmr1* function as an inhibitor of translation. Thus, reduction of *RanBPM* relieves the remaining *dfmr1* function thereby partially suppressing the NMJ overgrowth. In our studies we used *dfmr1* mutations that, while causing similar NMJ phenotypes, were either a complete loss *(dfmr1^Δ50M^*) or a partial loss of function *(dfmr1^EP3517^*). Yet, the *RanBPM^k05201^* mutation suppressed the *dfmr1* NMJ phenotype to the same extent (Figure 6). These observations suggest, as a more likely model, that *RanBPM* function is required in a dose-dependent fashion for NMJ development and not directly for *dfmr1* function. Taken together, the work reported here support the hypothesis that the underlying cause of the *RanBPM* behavioral phenotypes is lack of gene function in the MB neurons, and point to a novel role for this structure in larval behavior.

## Supporting Information

Figure S1 Locomotion of RanBPM[k05201] mutant and RanBPMrevertant in constant dark. Representative perimeter stacks generated using DIAS depicting larval locomotion during 60 sec in the absence of light transition under safelight.
(2.69 MB TIF)

Figure S2 RanBPM[k05201] third instar foraging larvae are smaller than control larvae. DIAS was used to measure the long axis of larval images. Under the DIAS function “measure” we used “simple length” to measure the number of pixels along the anterior posterior axis of individual larvae. The “scale” function was used to obtain the scale factor value employed to convert pixels into µm. RanBPM[k05201] mutants are significantly smaller than all control larvae of the same developmental stage. yw is significantly smaller than OR but not RanBPM revertant, *p<0.05, N = 10, (ANOVA, F(3, 36)  = 54.943, p<0.0001).
(6.03 MB TIF)

Figure S3 Contact chemosensory assay. The assay arena was divided into four quadrants. Opposing quadrants were filled with 1% agar in 1 M NaCl or in water. Larvae were placed in the center and allowed to migrate. Their distribution was determined at 10 and 15 min. Larvae that did not migrate more than 1 cm from the center of the plate were not included. The percentage of larvae present in the non salt quadrants was plotted. All genotypes showed a non- random distribution between the salt non-salt quadrants. The preference of RanBPM mutants for the non-salt quadrant at 10 min (x2 = 2.70, DF = 3, p<0.439) and 15 min (x2 = 3.84, DF = 3, p<0.279)is not significantly different from that of the control genotypes (OR, yw, RanBPM revertant). N≥50.
(5.03 MB TIF)

Figure S4 RanBPM is not required for differentiation and/or maintenance of various larval neurons.Confocal micrographs of RanBPM[k05201] larval brains labeled with various reporters and antibodies. In all panels the symbol ' (prime) indicates homozygous mutant specimens to the right of control heterozygous. Targeted expression of GFP under the control of the 247-GAL4 driver (247:GFP) in RanBPM mutants shows that the structure of MB neurons and neuropil is largely intact, although the volume appears reduced (green, A, A', B, B'). This is also true for the pattern of peptidergic neurons revealed by the expression of GFP under the control of the 386-GAL4 driver (386-GFP, green, C, C'). Double labeling of 386-GFP specimens with FasII antibody commonly used to label the MB neuropil area indicates that MB structure in these mutants is largely unaltered at this level of resolution but the volume may be reduced (red, D, D'). The FMRF amide antibody detects a subset of FMRF amide like peptides that contain a common RF amide sequence on their C-terminal. Included in this group is sNPF, the only known peptide to be expressed in the Kenyon cells. The expected pattern of expression detected by FMRF amide antibody is seen in the whole CNS (E-E'), MB neuropil area (F, F') and Kenyon cells (G, G'). 5-HT labeling reveals a stereotypical segmental pattern of neuronal cell bodies in RanBPM mutant, indistinguishable from control (I and I'), however cell counts revealed a small but significant reduction in the cell number (Table in J). Consistent with the observation that the MB neuropil area is reduced in these mutants we found that the 5HT arborization typically found in the larval optic neuropil is reduced in RanBPM[K05201] mutants (arrowhead in I and I'). All images except for those shown in panels B and B' are projections of Z stacks of 20 sections at 1 to 2 µm intervals.
(9.80 MB TIF)

Figure S5 RanBPM is not expressed in proliferating cells or glia. Confocal micrographs of third instar larval CNS double labeled with anti-RanBPM (green) and anti-phosphoH3 (red, A–F), or the glial marker anti-Repo (red, G–L). Boxed areas in B and K are magnified in D–F and J–K respectively and highlight RanBPM expression in the area of the lobes where the MB neurons are located. Co-localization was not detected for anti-Repo labeling (G–L). Apparent co-expression in A–F is due to both primary antibodies being detected by the same secondary (Cy3-conjugated goat anti-rabbit). We concluded that RanBPM is not expressed in actively dividing cells.
(7.04 MB TIF)

Table S1 Lethal complementation test for RanBPM mutants.
(0.04 MB DOC)

Table S2 Fraction of RanBPM mutant larvae that ingested food in 30 min.
(0.04 MB DOC)

Table S3 Lethality of RanBPM[k05201] mutants expressing either RanBPM isoform under the regulation of different GAL4 drivers.
(0.04 MB DOC)

Movie S1 Larval behavior during the food attraction assay: revertant control. RanBPM revertant larvae were placed outside the yeast drop and left for 1.5 hours. Larval behavior was captured during a representative assay and is shown here as an mp4 movie. The vast majority of the revertant control larvae moved into the food and remained there for the duration of the assay.
(2.19 MB MP4)

Movie S2 Larval behavior during the food attraction assay: RanBPM[K05201]. RanBPM mutant larvae were placed outside the yeast drop and left for 1.5 hours. Larval behavior was captured during a representative assay and is shown here as an mp4 movie. Most of the RanBPM mutant larvae (26 out 30) moved away from the original location but only a small fraction entered and/or remained in the food for the duration of the assay.
(2.68 MB MP4)
