# Supplementary figures and images for: The Drosophila Gene RanBPM Functions in the Mushroom Body to Regulate Larval Behavior
Source: PLoS One. 2010 May 14;5(5):e10652. doi: 10.1371/journal.pone.0010652 (PMC2871054; doi:10.1371/journal.pone.0010652)

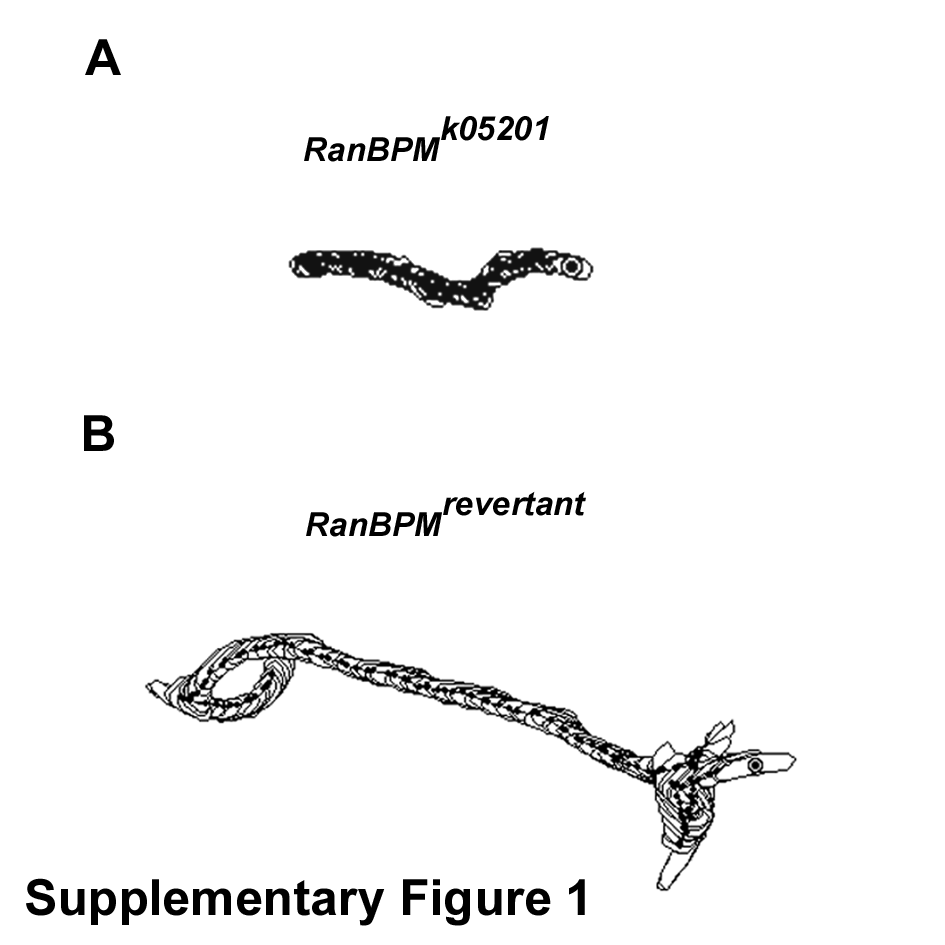

Supplement: Figure S1 — Locomotion of RanBPM[k05201] mutant and RanBPMrevertant in constant dark. Representative perimeter stacks generated using DIAS depicting larval locomotion during 60 sec in the absence of light transition under safelight. (2.69 MB TIF) [file pone.0010652.s001.tif]

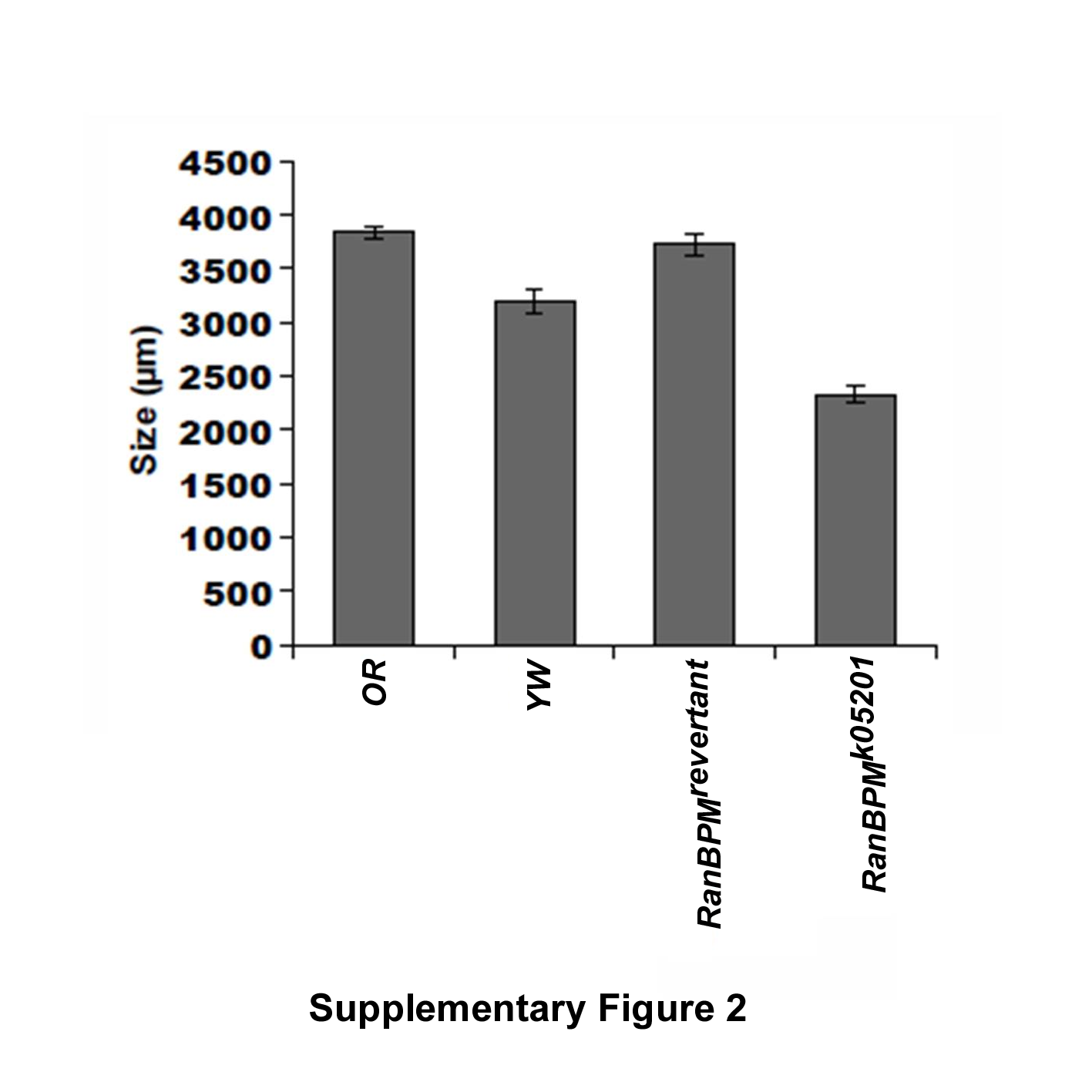

Supplement: Figure S2 — RanBPM[k05201] third instar foraging larvae are smaller than control larvae. DIAS was used to measure the long axis of larval images. Under the DIAS function “measure” we used “simple length” to measure the number of pixels along the anterior posterior axis of individual larvae. The “scale” function was used to obtain the scale factor value employed to convert pixels into µm. RanBPM[k05201] mutants are significantly smaller than all control larvae of the same developmental stage. yw is significantly smaller than OR but not RanBPM revertant, *p<0.05, N = 10, (ANOVA, F(3, 36) = 54.943, p<0.0001). (6.03 MB TIF) [file pone.0010652.s002.tif]

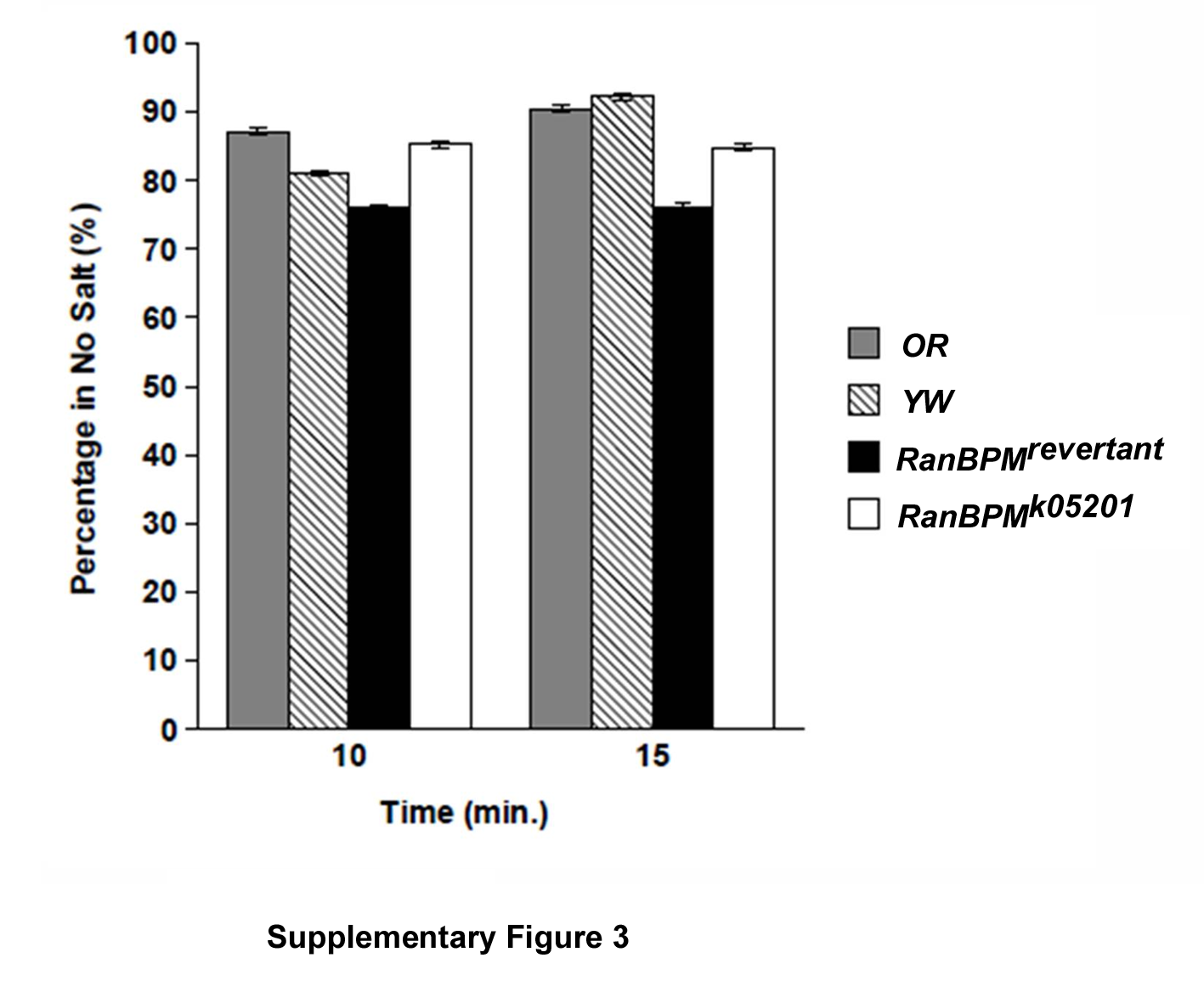

Supplement: Figure S3 — Contact chemosensory assay. The assay arena was divided into four quadrants. Opposing quadrants were filled with 1% agar in 1 M NaCl or in water. Larvae were placed in the center and allowed to migrate. Their distribution was determined at 10 and 15 min. Larvae that did not migrate more than 1 cm from the center of the plate were not included. The percentage of larvae present in the non salt quadrants was plotted. All genotypes showed a non- random distribution between the salt non-salt quadrants. The preference of RanBPM mutants for the non-salt quadrant at 10 min (x2 = 2.70, DF = 3, p<0.439) and 15 min (x2 = 3.84, DF = 3, p<0.279)is not significantly different from that of the control genotypes (OR, yw, RanBPM revertant). N≥50. (5.03 MB TIF) [file pone.0010652.s003.tif]

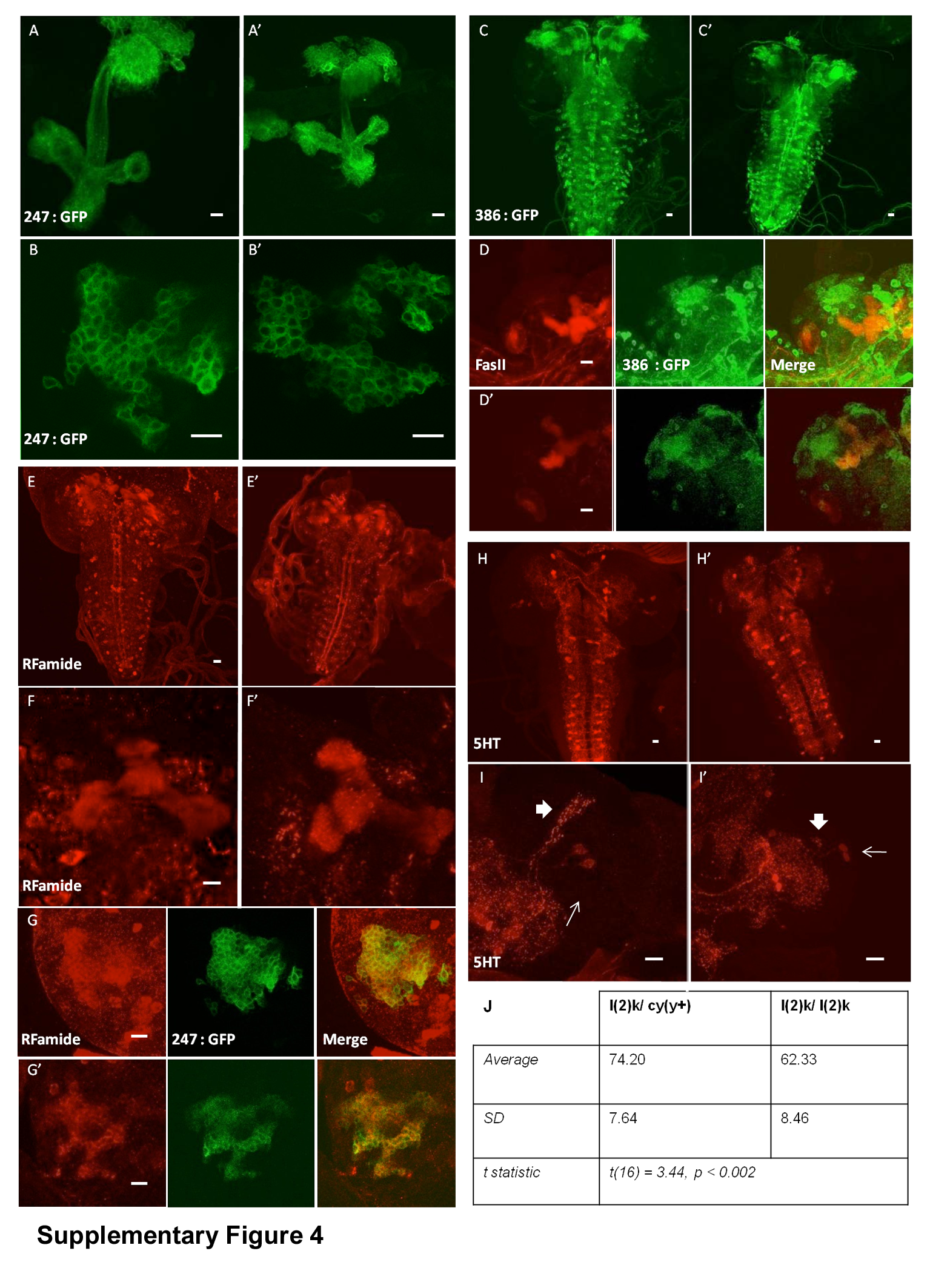

Supplement: Figure S4 — RanBPM is not required for differentiation and/or maintenance of various larval neurons.Confocal micrographs of RanBPM[k05201] larval brains labeled with various reporters and antibodies. In all panels the symbol ' (prime) indicates homozygous mutant specimens to the right of control heterozygous. Targeted expression of GFP under the control of the 247-GAL4 driver (247:GFP) in RanBPM mutants shows that the structure of MB neurons and neuropil is largely intact, although the volume appears reduced (green, A, A', B, B'). This is also true for the pattern of peptidergic neurons revealed by the expression of GFP under the control of the 386-GAL4 driver (386-GFP, green, C, C'). Double labeling of 386-GFP specimens with FasII antibody commonly used to label the MB neuropil area indicates that MB structure in these mutants is largely unaltered at this level of resolution but the volume may be reduced (red, D, D'). The FMRF amide antibody detects a subset of FMRF amide like peptides that contain a common RF amide sequence on their C-terminal. Included in this group is sNPF, the only known peptide to be expressed in the Kenyon cells. The expected pattern of expression detected by FMRF amide antibody is seen in the whole CNS (E-E'), MB neuropil area (F, F') and Kenyon cells (G, G'). 5-HT labeling reveals a stereotypical segmental pattern of neuronal cell bodies in RanBPM mutant, indistinguishable from control (I and I'), however cell counts revealed a small but significant reduction in the cell number (Table in J). Consistent with the observation that the MB neuropil area is reduced in these mutants we found that the 5HT arborization typically found in the larval optic neuropil is reduced in RanBPM[K05201] mutants (arrowhead in I and I'). All images except for those shown in panels B and B' are projections of Z stacks of 20 sections at 1 to 2 µm intervals. (9.80 MB TIF) [file pone.0010652.s004.tif]

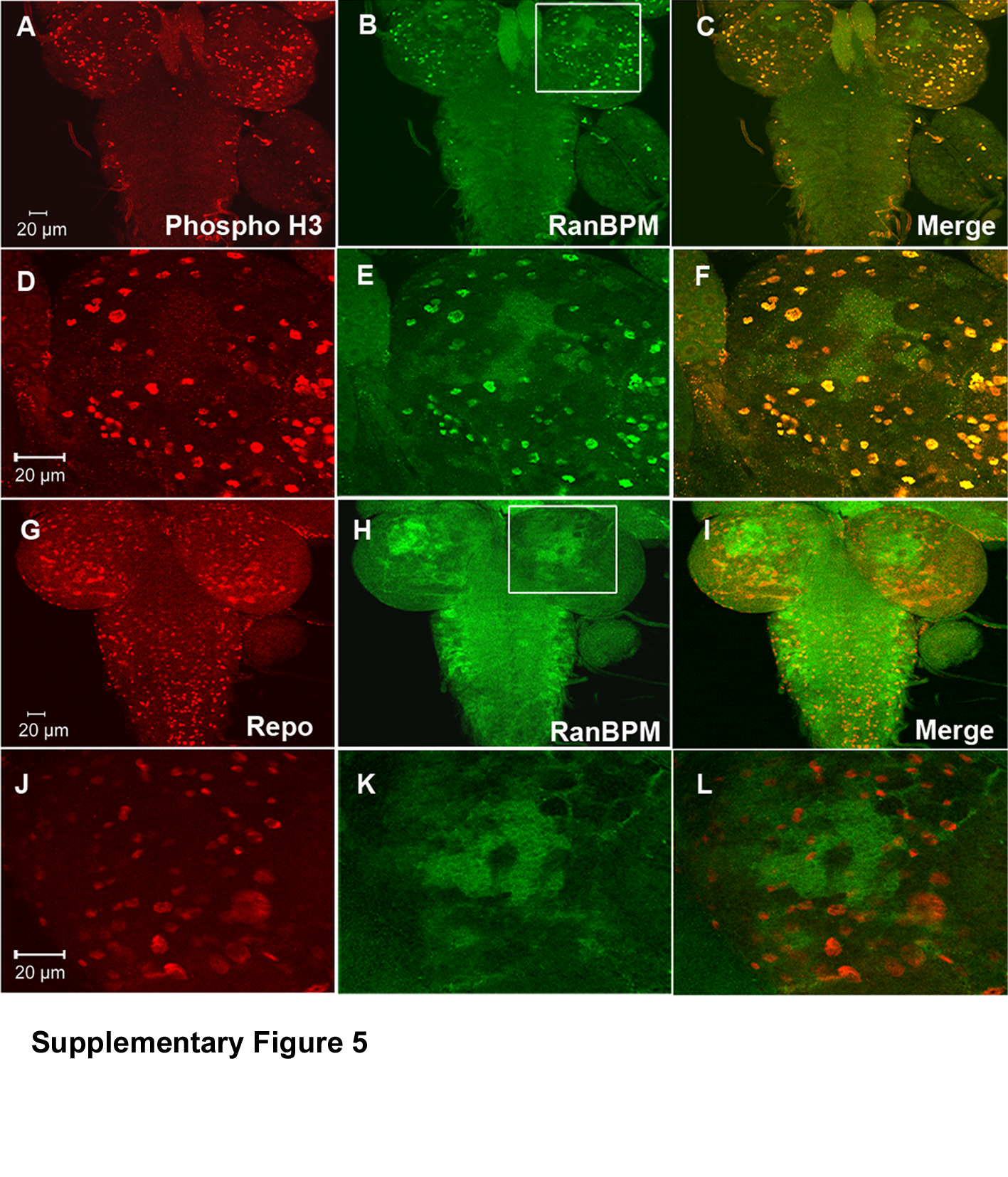

Supplement: Figure S5 — RanBPM is not expressed in proliferating cells or glia. Confocal micrographs of third instar larval CNS double labeled with anti-RanBPM (green) and anti-phosphoH3 (red, A–F), or the glial marker anti-Repo (red, G–L). Boxed areas in B and K are magnified in D–F and J–K respectively and highlight RanBPM expression in the area of the lobes where the MB neurons are located. Co-localization was not detected for anti-Repo labeling (G–L). Apparent co-expression in A–F is due to both primary antibodies being detected by the same secondary (Cy3-conjugated goat anti-rabbit). We concluded that RanBPM is not expressed in actively dividing cells. (7.04 MB TIF) [file pone.0010652.s005.tif]
