# Supplementary material for: The Drosophila Gene RanBPM Functions in the Mushroom Body to Regulate Larval Behavior
Source: PLoS One. 2010 May 14;5(5):e10652. doi: 10.1371/journal.pone.0010652 (PMC2871054; doi:10.1371/journal.pone.0010652)
Supplement: Table S1 — Lethal complementation test for RanBPM mutants. (0.04 MB DOC) [file pone.0010652.s006.doc]

**Table S1.**

**Lethal complementation test for *RanBPM* mutants.**

|  | *RanBPM ^k05201^* | *RanBPM ^s135^* | *RanBPM ^ts7^* |
| --- | --- | --- | --- |
| *RanBPM ^k05201^* | 100 | 100 | 22^*^ |
| *RanBPM ^s135^* |  | 100 | 88.75^*^ |
| *RanBPM ^ts7^* |  |  | 100 |

Data are displayed as mean % lethality, ie. fraction of expected progeny that did not survive to adulthood (at least 200 flies screened). *RanBPM ^k05201^* and *RanBPM ^s135^* behave as a strict recessive lethal alleles. The *RanBPM ^ts7^* allele behaves as a hypomorphic allele that carries a background lethal. All escapers showed a spread wing phenotype similar to that of *Dichaete* (D) mutants.
