# Supplementary material for: The Drosophila Gene RanBPM Functions in the Mushroom Body to Regulate Larval Behavior
Source: PLoS One. 2010 May 14;5(5):e10652. doi: 10.1371/journal.pone.0010652 (PMC2871054; doi:10.1371/journal.pone.0010652)
Supplement: Table S2 — Fraction of RanBPM mutant larvae that ingested food in 30 min. (0.04 MB DOC) [file pone.0010652.s007.doc]

**Table S2.**

**Fraction of *RanBPM* mutant larvae that ingested food in 30 min.**

*RanBPM^s135^RanBPM^ts7^RanBPM^k05201^*14.33 ± 2.0383.33 ± 2.91*RanBPM^s135^*14 ± 1.7381.33 ± 3.38*RanBPM^ts7^*79.67±4.06

Data are displayed as mean % ± S.E.M. Each experiment was performed three times and in each round at least 18 larvae were scored.
