# Supplementary material for: The Drosophila Gene RanBPM Functions in the Mushroom Body to Regulate Larval Behavior
Source: PLoS One. 2010 May 14;5(5):e10652. doi: 10.1371/journal.pone.0010652 (PMC2871054; doi:10.1371/journal.pone.0010652)
Supplement: Table S3 — Lethality of RanBPM[k05201] mutants expressing either RanBPM isoform under the regulation of different GAL4 drivers. (0.04 MB DOC) [file pone.0010652.s008.doc]

**Table S3.**

**Lethality of *RanBPM ^k05201^* mutants expressing either *RanBPM***

**isoform under the regulation of different *GAL4* drivers.**

| *RanBPM ^k05201^;GAL4* | *RanBPM ^k05201^; UAS-RanBPM;* | |
| --- | --- | --- |
|  | *Long* | *Short* |
| *elav-GAL4* | 86.31** | 100 |
| *247-GAL4* | 100 | 100 |
| *386-GL4* | 100 | 100 |
| *Dmef2-GAL4* | 97.18** | 100 |

Data are displayed as mean % lethality, i.e. fraction of expected progeny that did not survive to adulthood (at least 200 flies screened). 100% represents no rescue, 0% represents complete rescue.

** All survivors display a Dichaete-like phenotype of spread wing undistinguishable from that screen in escapers carrying a less severe heteroallelic combination (Supplementary. Table 1.)
